# Supplementary material for: Quality of Life in Rectal Cancer Treatments: An Updated Systematic Review of Randomized Controlled Trials (2013–2023)
Source: Cancers (Basel). 2025 Jul 11;17(14):2310. doi: 10.3390/cancers17142310 (PMC12294010; doi:10.3390/cancers17142310)
Supplement: Supplementary file 1 [file cancers-17-02310-s001.zip › cancers-3641623-supplementary.pdf]

Supplementary Materials – Index

- **Search Strategy** – pp. 2–3
- **AMSTAR 2 Assessment** – pp. 4–7
- **Table S1.** Studies on Surgical Interventions Included in the Review – pp. 8–10
- **Table S2.** Studies on Pre-/Post-Chemotherapy and/or Radiotherapy Included in the Review – pp. 11–14
- **Table S3.** Studies on Patient Care Strategies Included in the Review – pp. 15–17
- **Figure S1.** Risk of Bias Assessment (RoB 2) for Surgical Intervention Studies – pp. 19
- **Figure S2.** Risk of Bias Assessment (RoB 2) for Pre- and/or Post-Chemotherapy and/or Radiotherapy Studies – pp. 20
- **Figure S3.** Risk of Bias Assessment (RoB 2) for Patient Care Strategy Studies – pp. 21

Search Strategy

Computer-assisted search code according to Embase & Medline & Cochrane databases.

Source		Selection code
Embase & Medline & Cochrane (via Ovid)	1	rectal.mp. [mp=ti, ot, ab, fx, sh, hw, kw, tx, ct, tn, dm, mf, dv, kf, dq, bt, nm, ox, px, rx, an, ui, sy, ux, mx]
	2	cancer.mp. [mp=ti, ot, ab, fx, sh, hw, kw, tx, ct, tn, dm, mf, dv, kf, dq, bt, nm, ox, px, rx, an, ui, sy, ux, mx]
	3	neoplasm.mp. [mp=ti, ot, ab, fx, sh, hw, kw, tx, ct, tn, dm, mf, dv, kf, dq, bt, nm, ox, px, rx, an, ui, sy, ux, mx]
	4	quality of life.mp. [mp=ti, ot, ab, fx, sh, hw, kw, tx, ct, tn, dm, mf, dv, kf, dq, bt, nm, ox, px, rx, an, ui, sy, ux, mx]
	5	functional outcomes.mp. [mp=ti, ot, ab, fx, sh, hw, kw, tx, ct, tn, dm, mf, dv, kf, dq, bt, nm, ox, px, rx, an, ui, sy, ux, mx]
	6	surgery.mp. [mp=ti, ot, ab, fx, sh, hw, kw, tx, ct, tn, dm, mf, dv, kf, dq, bt, nm, ox, px, rx, an, ui, sy, ux, mx]
	7	treatment.mp. [mp=ti, ot, ab, fx, sh, hw, kw, tx, ct, tn, dm, mf, dv, kf, dq, bt, nm, ox, px, rx, an, ui, sy, ux, mx]
	8	therapy.mp. [mp=ti, ot, ab, fx, sh, hw, kw, tx, ct, tn, dm, mf, dv, kf, dq, bt, nm, ox, px, rx, an, ui, sy, ux, mx]
	9	chemotherapy.mp. [mp=ti, ot, ab, fx, sh, hw, kw, tx, ct, tn, dm, mf, dv, kf, dq, bt, nm, ox, px, rx, an, ui, sy, ux, mx]
	10	radiotherapy.mp. [mp=ti, ot, ab, fx, sh, hw, kw, tx, ct, tn, dm, mf, dv, kf, dq, bt, nm, ox, px, rx, an, ui, sy, ux, mx]
	11	HRQoL.mp. [mp=ti, ot, ab, fx, sh, hw, kw, tx, ct, tn, dm, mf, dv, kf, dq, bt, nm, ox, px, rx, an, ui, sy, ux, mx]
	12	surgical intervention.mp. [mp=ti, ot, ab, fx, sh, hw, kw, tx, ct, tn, dm, mf, dv, kf, dq, bt, nm, ox, px, rx, an, ui, sy, ux, mx]
	13	patient care.mp. [mp=ti, ot, ab, fx, sh, hw, kw, tx, ct, tn, dm, mf, dv, kf, dq, bt, nm, ox, px, rx, an, ui, sy, ux, mx]

	**14**	continuity of care.mp. [mp=ti, ot, ab, fx, sh, hw, kw, tx, ct, tn, dm, mf, dv, kf, dq, bt, nm, ox, px, rx, an, ui, sy, ux, mx]
	**15**	2 or 3
	**16**	4 or 5 or 11
	**17**	6 or 7 or 8 or 9 or 10 or 12 or 13 or 14
	**18**	6 or 7 or 8 or 9 or 10 or 12 or 13 or 14
	**19**	1 and 15 and 16 and 17 and 18
	**20**	limit 19 to randomized controlled trial [Limit not valid in CCTR,CDSR; records were retained]
	**21**	limit 20 to english language [Limit not valid in CDSR; records were retained]
	**22**	limit 21 to human [Limit not valid in CCTR,CDSR; records were retained]
	**23**	limit 22 to english
	**24**	limit 23 to yr="2013 - 2023"
	**25**	remove duplicates from 24

Computer-assisted search code according to Scopus database

TITLE-ABS-KEY (("rectal cancer" OR "rectal neoplasm*") AND ("quality of life" OR HRQoL OR "functional outcome*" OR "patient-reported outcomes") AND ("surgery" OR "surgical treatment" OR "surgical intervention" OR "chemotherapy" OR "radiotherapy" OR "therapy" OR "treatment" OR "patient care" OR "continuity of care") AND ("RCT" OR "randomized controlled trial”)) limit to English and 2013-2023 352 documents

AMSTAR 2: a critical appraisal tool for systematic reviews that include randomised or non-randomised studies of healthcare interventions, or both

1. Did the research questions and inclusion criteria for the review include the components of PICO?

For Yes:	Optional (recommended)	
☒ Population<br>☒ Intervention<br>☒ Comparator group<br>☒ Outcome	☐ Timeframe for follow-up	☒ Yes<br>☐ No

2. Did the report of the review contain an explicit statement that the review methods were established prior to the conduct of the review and did the report justify any significant deviations from the protocol?

For Partial Yes:	For Yes:	
The authors state that they had a written protocol or guide that included ALL the following:<br>☒ review question(s)<br>☒ a search strategy<br>☒ inclusion/exclusion criteria<br>☒ a risk of bias assessment	As for partial yes, plus the protocol should be registered and should also have specified:<br>☐ a meta-analysis/synthesis plan, if appropriate, *and*<br>☐ a plan for investigating causes of heterogeneity<br>☐ justification for any deviations from the protocol	☐ Yes<br>☒ Partial Yes<br>☐ No

3. Did the review authors explain their selection of the study designs for inclusion in the review?

For Yes, the review should satisfy ONE of the following:	
☒ *Explanation for* including only RCTs<br>☐ OR *Explanation for* including only NRSI<br>☐ OR *Explanation for* including both RCTs and NRSI	☒ Yes<br>☐ No

4. Did the review authors use a comprehensive literature search strategy?

For Partial Yes (all the following):	For Yes, should also have (all the following):	
☒ searched at least 2 databases (relevant to research question)<br>☐ provided key word and/or search strategy<br>☐ justified publication restrictions (e.g. language)	☒ searched the reference lists / bibliographies of included studies<br>☒ searched trial/study registries<br>☒ included/consulted content experts in the field<br>☒ where relevant, searched for grey literature<br>☒ conducted search within 24 months of completion of the review	☒ Yes<br>☐ Partial Yes<br>☐ No

5. Did the review authors perform study selection in duplicate?

For Yes, either ONE of the following:	
☒ at least two reviewers independently agreed on selection of eligible studies and achieved consensus on which studies to include<br>☐ OR two reviewers selected a sample of eligible studies <u>and</u> achieved good agreement (at least 80 percent), with the remainder selected by one reviewer.	☒ Yes<br>☐ No

AMSTAR 2: a critical appraisal tool for systematic reviews that include randomised or non-randomised studies of healthcare interventions, or both

6. Did the review authors perform data extraction in duplicate?

For Yes, either ONE of the following:

- [x] at least two reviewers achieved consensus on which data to extract from included studies
- [ ] OR two reviewers extracted data from a sample of eligible studies and achieved good agreement (at least 80 percent), with the remainder extracted by one reviewer.

- [x] Yes
- [ ] No

7. Did the review authors provide a list of excluded studies and justify the exclusions?

For Partial Yes:

- [x] provided a list of all potentially relevant studies that were read in full-text form but excluded from the review

For Yes, must also have:

- [ ] Justified the exclusion from the review of each potentially relevant study

- [ ] Yes
- [x] Partial Yes
- [ ] No

8. Did the review authors describe the included studies in adequate detail?

For Partial Yes (ALL the following):

- [x] described populations
- [x] described interventions
- [x] described comparators
- [x] described outcomes
- [x] described research designs

For Yes, should also have ALL the following:

- [ ] described population in detail
- [ ] described intervention in detail (including doses where relevant)
- [ ] described comparator in detail (including doses where relevant)
- [ ] described study's setting
- [ ] timeframe for follow-up

- [ ] Yes
- [x] Partial Yes
- [ ] No

9. Did the review authors use a satisfactory technique for assessing the risk of bias (RoB) in individual studies that were included in the review?

RCTs

For Partial Yes, must have assessed RoB from

- [ ] unconcealed allocation, *and*
- [ ] lack of blinding of patients and assessors when assessing outcomes (unnecessary for objective outcomes such as all-cause mortality)

For Yes, must also have assessed RoB from:

- [x] allocation sequence that was not truly random, *and*
- [x] selection of the reported result from among multiple measurements or analyses of a specified outcome

- [x] Yes
- [ ] Partial Yes
- [ ] No
- [ ] Includes only NRSI

NRSI

For Partial Yes, must have assessed RoB:

- [ ] from confounding, *and*
- [ ] from selection bias

For Yes, must also have assessed RoB:

- [ ] methods used to ascertain exposures and outcomes, *and*
- [ ] selection of the reported result from among multiple measurements or analyses of a specified outcome

- [ ] Yes
- [ ] Partial Yes
- [ ] No
- [x] Includes only RCTs

10. Did the review authors report on the sources of funding for the studies included in the review?

For Yes

- [x] Must have reported on the sources of funding for individual studies included in the review. Note: Reporting that the reviewers looked for this information but it was not reported by study authors also qualifies

- [x] Yes
- [ ] No

11. If meta-analysis was performed did the review authors use appropriate methods for statistical combination of results?

RCTs
For Yes:

- ☐ The authors justified combining the data in a meta-analysis
  - ☐ AND they used an appropriate weighted technique to combine study results and adjusted for heterogeneity if present.
  - ☐ AND investigated the causes of any heterogeneity

☐ Yes
☐ No
☒ No meta-analysis conducted

For NRSI
For Yes:

- ☐ The authors justified combining the data in a meta-analysis
  - ☐ AND they used an appropriate weighted technique to combine study results, adjusting for heterogeneity if present
  - ☐ AND they statistically combined effect estimates from NRSI that were adjusted for confounding, rather than combining raw data, or justified combining raw data when adjusted effect estimates were not available
  - ☐ AND they reported separate summary estimates for RCTs and NRSI separately when both were included in the review

☐ Yes
☐ No
☒ No meta-analysis conducted

12. If meta-analysis was performed, did the review authors assess the potential impact of RoB in individual studies on the results of the meta-analysis or other evidence synthesis?

For Yes:

- ☐ included only low risk of bias RCTs
- ☐ OR, if the pooled estimate was based on RCTs and/or NRSI at variable RoB, the authors performed analyses to investigate possible impact of RoB on summary estimates of effect.

☐ Yes
☐ No
☒ No meta-analysis conducted

13. Did the review authors account for RoB in individual studies when interpreting/ discussing the results of the review?

For Yes:

- ☐ included only low risk of bias RCTs
- ☒ OR, if RCTs with moderate or high RoB, or NRSI were included the review provided a discussion of the likely impact of RoB on the results

☒ Yes
☐ No

14. Did the review authors provide a satisfactory explanation for, and discussion of, any heterogeneity observed in the results of the review?

For Yes:

- ☐ There was no significant heterogeneity in the results
- ☒ OR if heterogeneity was present the authors performed an investigation of sources of any heterogeneity in the results and discussed the impact of this on the results of the review

☒ Yes
☐ No

15. If they performed quantitative synthesis did the review authors carry out an adequate investigation of publication bias (small study bias) and discuss its likely impact on the results of the review?

For Yes:

- ☐ performed graphical or statistical tests for publication bias and discussed the likelihood and magnitude of impact of publication bias

☐ Yes
☐ No
☒ No meta-analysis conducted

AMSTAR 2: a critical appraisal tool for systematic reviews that include randomised or non-randomised studies of healthcare interventions, or both

16. **Did the review authors report any potential sources of conflict of interest, including any funding they received for conducting the review?**

For Yes:	
☒ The authors reported no competing interests OR	☒ Yes
☐ The authors described their funding sources and how they managed potential conflicts of interest	☐ No

To cite this tool: Shea BJ, Reeves BC, Wells G, Thuku M, Hamel C, Moran J, Moher D, Tugwell P, Welch V, Kristjansson E, Henry DA. AMSTAR 2: a critical appraisal tool for systematic reviews that include randomised or non-randomised studies of healthcare interventions, or both. BMJ. 2017 Sep 21;358:j4008.

Table S1. Articles on surgical interventions included in the review

Table S1. Articles on surgical interventions included in the review (continued)											
**Author, year (Ref.)**	**Study Design, Country**	**Duration of study**	**Population**	**Intervention**	**Number of Patients**	**Sex (M, %)**	**Distance from the av (cm)**	**Age (y)**	**Questionnaire(s)**	**Times of assessment**	**Key Results**
**Gadan et al, 2017 (20)**	Multicenter, Sweden	Dec 1999-June 2005	To compare HRQoL after temporary ileostomy vs. no ileostomy in patients undergoing LAR	Temporary ileostomy (n=41) vs no ileostomy (n=46)	87	Temporary ileostomy group:25(61)<br>No ileostomy group: 24(52.2)<br>N(%)	Temporary ileostomy group:5(2.5-7)<br>No ileostomy group: 5(3-7)<br>Median(range)	Temporary ileostomy group:62(32-82)<br>No ileostomy group:66(43-84)<br>Median (range)	EQ-5D-3L	12 y after the operation	Worse self-reported HRQoL in those with major LARS
**Jayne et al, 2017 (21)**	Multicenter, 10 counties	Jan 2011-Sept 2014	To compare HRQoL after robotic vs. laparoscopic surgery in patients undergoing LAR	Robotic Surgery (n=237) vs Laparoscopic surgery (n=234)	471	Robotic group:161(67.9)<br>Laparoscopic group: 159(67.9)<br>N(%)	Robotic group:<br>11-15 :71(30.1)<br>6-10: 107(45.3)<br>0-5: 57(24.2)<br>Missing : 1(0.4)<br>Laparoscopic group: 11-15 69(30)<br>6-10: 99(43)<br>0-5: 61(26.5)<br>Missing: 1(0.4)<br>N(%)	Robotic group:64.4(10.9)<br>Laparoscopic group:65.5(11.9)<br>Mean (SD)	SF 36	30 days, 6mo after the operation	No difference
**Kim et al, 2018 (22)**	Single center, South Korea	Feb 2012-March 2015	To compare HRQoL after robotic vs. laparoscopic surgery in patients undergoing LAR	Robotic Surgery (n=66) vs Laparoscopic surgery (n=73)	139	Robotic group:51(77.3)<br>Laparoscopic group: 52(71.2)<br>N(%)	Robotic group:<br>≤5:33(50)<br>>5:33(50)<br>Laparoscopic group:<br>≤5:35(48)<br>>5:38(52)<br>N(%)	Robotic group:60.4(9.7)<br>Control Laparoscopic group:59.7(11.7)<br>Mean (SD)	SF 36	3 we, 3 mo, 12 mo after the operation	No difference
**Park et al, 2018 (23)**	Multicenter, Denmark and Sweden	Feb 2011- Nov 2015	To compare HRQoL after early vs. late ileostomy closure after LAR	Early ileostomy closure (8-13 days after surgery) (n=55) vs late ileostomy closure (>12 we after surgery) (n=57)	112	Early ileostomy closure:<br>24(43.6)<br>Late ileostomy closure: 36(63.1)<br>N(%)	Early ileostomy closure:<br>5-9: 27(49)<br>10-15: 27(49)<br>>15: 1 (2)<br>Late ileostomy closure: 5-9: 24(42)<br>10-15: 33(58)<br>>15: 0(0)<br>N(%)	Early ileostomy closure:<br>67(36-82)<br>Late ileostomy closure: 67(39-81)<br>Median (Range)	EORTC-QLQ CR29<br>EORTC-QLQ-C30<br>SF36	3, 6, 12mo after the operation	No difference

Table S1. Articles on surgical interventions included in the review (continued)											
**Author, year (Ref.)**	**Study Design, Country**	**Duration of study**	**Population**	**Intervention**	**Number of Patients**	**Sex (M, %)**	**Distance from the av (cm)**	**Age (y)**	**Questionnaire(s)**	**Times of assessment**	**Key Results**
**Parc at el, 2019 (24)**	Multicenter, France, Germany, USA, Australia	2007-2009	To compare HRQoL after colonic j-pouch vs. side-to-end anastomosis	CJP (n=80) vs. SEA (n=87)<br><br>*SEA: Side-to-End anastomosis*<br>*CJP: Colon J-Pouch anastomosis*	167	CJP:59(73.8)<br>SEA:52(59.8)<br><br>N(%)	N/A	CJP:60.2(9.7)<br>SEA:59.6(10.6)<br><br>Mean(SD)	SF12, FACT-C	6, 12 and 24mo after surgery	No difference
**Ribi et al, 2019 (25)**	Multicenter, Switzerland	Sep 2005-May 2014	To compare HRQoL after colonic j-pouch vs. side-to-end vs. straight colorectal anastomosis	CJP (n=63)vs. SEA (n=95) vs. SCA (n=99)<br><br>*SEA: Side-to-End anastomosis*<br>*CJP: Colon J-Pouch anastomosis*<br>*SCA: Straight Colorectal anastomosis*	257	CJP:38 (60.3)<br>SEA:62(65.3)<br>SCA:67(67.7)<br><br>N(%)	CJP: 6.0 [1.0, 11.0]; SEA: 7.0 [1.0, 15.0]; SCA: 5.0 [1.0, 12.0]<br><br>Median (IQR)	CJP:68.6 (30.9,85.5);<br>SEA:67.2(32.3, 88.9);<br>SCA:66.3(32.3, 90.9)<br><br>Median (IQR)	FACT-C	6, 12, 28 and 24 mo after surgery	No difference
**Gavaruzzi et al, 2020 (26)**	Multicenter, Italy	Oct 2009- Feb 2016	To compare HRQoL after colonic j-pouch vs. straight colorectal anastomosis	CJP (n=161) vs. SCA (n=158)<br><br>*CJP: Colon J-Pouch anastomosis*<br>*SCA: Straight Colorectal anastomosis*	319	CJP: 89(55.2)<br>SCA:95(60.1)<br><br>N(%)	CJP: 7(5-9)<br>SCA:8 (6-9)<br><br>Median (IQR)	CJP:66(58-72)<br>SCA:63(55-72)<br><br>Median (IQR)	EORTC QLC C30<br>EORTC QLC C38	6, 12, 24mo after the operation	No difference
**Bach et al, 2020 (27)**	Multicenter, UK	Feb 2012- Dec 2014	To compare HRQoL after organ preservation (LE) vs. Radical surgery in cT2, or lower RC, N0, M0, who underwent short course RT	Organ preservation (LE) (n=27) vs. Radical surgery (n=28)	56	Organ preservation group:19(70)<br>Radical surgery group: 17(61)<br><br>N(%)	Organ preservation group:6(4-8)<br>Radical surgery group:6(5-7)<br><br>Median (IQR)	Organ preservation group:65(52-79)<br>Radical surgery group:65(49-83)<br><br>Median (IQR)	EORTC QLC C30<br>EORTC QLC CR29	3, 6, 12, 24 and 36 mo after the operation	Worse QoL in the areas of health anxiety, role, and social function for the organ preservation group

Table S1. Articles on surgical interventions included in the review (continued)											
**Author, year (Ref.)**	**Study Design, Country**	**Duration of study**	**Population**	**Intervention**	**Number of Patients**	**Sex (M, %)**	**Distance from the av (cm)**	**Age (y)**	**Questionnaire(s)**	**Times of assessment**	**Key Results**
**Elsner et al, 2021 (28)**	Multicenter, Switzerland	Nov 2007-Mar 2014	To compare HRQoL after early vs. late ileostomy closure after LAR	Early ileostomy closure (2 we) (n=37) vs. Late ileostomy closure (12we) (n=34)	71	Early ileostomy closure: 21(56.7) Late ileostomy closure group: 26(76.4) N(%)	Early ileostomy closure group:3(1-5) Late ileostomy closure group: 2.7(1-5) Median (Range)	Early ileostomy closure group: 67(41-88) Late ileostomy closure group: 67(48-87) Median (Range)	EORTC- QLQ-C30	6 we and 4 mo after surgery	No difference
**Dulskas et al, 2021 (29)**	Single Centre, Lithuania	Dec 2011-Dec 2017	To compare HRQoL after early vs. late ileostomy closure after LAR	Early ileostomy closure (30 days) (n=26) vs Late ileostomy closure (90 days) (n=25)	51	Early ileostomy closure group: 14(53.8) Late ileostomy closure group: 11 (44) N(%)	Early ileostomy closure group: Low 3(11.5) Medium 18(69.2) High 5(19.3) Late ileostomy closure group: Low 4(16) Medium 18(72) High 3(12) N(%)	Early ileostomy closure group: 63(9.4) Late ileostomy closure group: 65(9.3) Median (IQR)	EORTC- QLQ-C30	36 mo	No difference
**Ellebaek et al, 2023 (30)**	Multicenter. Denmark	April 2011-Sep 2018	To compare HRQoL after early vs. late ileostomy closure after LAR	Early ileostomy closure (8-12 days) (n=77) vs Late ileostomy closure (>3 mo) (n=69)	146	Early ileostomy closure group: 53(69) Late ileostomy closure group: 52(75) N(%)	Early ileostomy closure group: 8.93(2.22) Late ileostomy closure group: 8.97(2.49) Mean(SD)	Early ileostomy closure group: 66.3(8.1) Late ileostomy closure group: 64.5(9.1) Mean(SD)	GIQLI	6 mo, 12 mo after the operation	No difference
**Ahmadi-Amoli et al, 2023 (31)**	Single Centre, Iran	2016-2020	To compare HRQoL after early vs. late ileostomy closure after LAR	Early ileostomy closure (2-3 we after the first two courses of adjuvant chemotherapy) (n=50) vs Late ileostomy closure (2-3 we after the last course of adjuvant chemotherapy) (n=54)	104	Early ileostomy closure group: 29(58) Late ileostomy closure group: 33(61.1) N(%)	Early ileostomy closure group: Low 15(30) Medium 30(60) High 5(10) Late ileostomy closure group: Low 24(44.4) Medium 26(48.1) High 4(7.4) Mean(SD)	Early ileostomy closure group: 63.2(1.70) Late ileostomy closure group: 63.18(1.49) Mean(SD)	SF 36	3, 12 mo after the operation	No difference
LAR: Low Anterior Resection; HRQoL: Health Related Quality of Life; LARS: Low Anterior Resection Syndrome; TME: Total Mesorectal Excision											

Table S2. Articles on pre- and/or post-CT and/or RT included in the review

Table S2. Articles on pre- and/or post-CT and/or RT included in the review											
**Author, year (Ref.)**	**Study Design, Country**	**Duration of study**	**Population**	**Intervention**	**Number of Patients**	**Sex (M, %)**	**Distance from the av (cm)**	**Age (y)**	**Questionnaire(s)**	**Times of assessment**	**Key Results**
**McLachlan et al, 2016 (32)**	Multicenter, Australia	2001-2006	To compare HRQoL after after Short- course RT vs. Long course CRT in patients with cT3N0-2M0	Short-course RT (n=143) vs Long-course CRT (n=154)	297	Short-course RT group: 102(71)<br>Long-course CRT group: 113(73)<br>N (%)	N/A	Short-course RT group: 63(26-80)<br>Long-course CRT group:64(29-82)<br>Median (range)	EORTC QLQ C30 – QLQC38	1, 2, 3, 6, 9, 12 mo after treatment	No difference
**Wiltink et al, 2016 (33)**	Multicenter, Duch	Jan 1996- Dec 1999	To compare HRQoL after short course RT followed by TME vs. TME alone in patients with LARC planned for surgery	Short-course RT (Study group, 5x5 Gy) followed by TME (n=241) vs TME alone (Control group, n=237)	478	Study group: 62.2<br>Control group: 55.3<br>%	Study group :<br>0-5: 29.2<br>5.1-10: 40.4<br>10.1-15: 30.4<br>Control group:<br><5: 32.2<br>5-10: 40.3<br>>10: 27.5<br>%	Study group: 62(43-95)<br>Control group:60(39-93)<br>Median (range)	EORTC QLQ C30 – QLQCR29	3, 6, 12, 18 , 24 mo, 5 y and 14 y	More bowel disfunction in the RT group
**Araujo et al, 2018 (34)**	Single center, Brazil	Jan, 2011 – Feb, 2013	To compare HRQoL after nCRT (Capecitabine) vs. nCRT (5-FU and Leucovorin) in patients with stage II and III RC planned for surgery	nCRT (Group 1, Capecitabine, n=31) vs. nCRT (Group 2, 5-FU and Leucovorin, n=30)	61	Group 1: 16(51.6)<br>Group 2: 17(56.7)<br>N(%)	Group 1: 4.9(2.8)<br>Group 2:3.7(2.4)<br>Mean(SD)	Group 1: 56.6(13.4)<br>Group 2: 60.5(8.6)<br>Mean(SD)	EORTC QLQ C30 – QLQCR38	6-8 we after nCRT, 30 days after surgery, after adjuvant CT, 1 year after the end of the treatment or stoma closure	No difference
**Sang Hong et al, 2019 (35)**	Multicenter, Korea	Nov 2008-Jun 2012	To compare HRQoL after adjuvant CT with Fl vs. adjuvant CT with FOLFOX in patients with ypStage II or III RC	Adjuvant CT with FL (fluorouracil and leucovorin) (Study group, n=161) vs Adjuvant CT with FOLFOX (Control group, n=160)	321	Study group: 116(72)<br>Control group: 118(73.8)<br>N (%)	Study:<br>≤4 cm: 45(28)<br>4 and ≤8 cm: 89(55.3)<br>>8: 27(16.8)<br>Control:<br>≤4 cm: 48(30)<br>4 and ≤8 cm: 81(50.6)<br>>8: 31(19.4)<br>N(%)	Study group: 54(25-79)<br>Control group: 55(27-81)<br>Median (range)	EORTC QLQ C30 – QLQCR38	2 mo, and at the end of treatment	No difference
**Van der Valk et al, 2019 (36)**	Multicenter, Duch	2004-2013	To compare HRQoL after adjuvant CT in patients with ypStage II or III RC who underwent preoperative (CT)RT	Adjuvant CT (Capecitabine, 8 courses) (Study group, n=115) vs Observation (Control group, n=111)	226	Study group: 67(58)<br>Control group: 76(68)<br>N (%)	N/A	Study group: 60.68<br>Control group:61.13<br>Mean	EORTC QLQ C30 – QLQCR38	1, 3, 6 and 12 mo after surgery	No difference

Table S2. Articles on pre- and/or post-CT and/or RT included in the review (continued)											
**Author, year (Ref.)**	**Study Design, Country**	**Duration of study**	**Population**	**Intervention**	**Number of Patients**	**Sex (M, %)**	**Distance from the av (cm)**	**Age (y)**	**Questionnaire(s)**	**Times of assessment**	**Key Results**
**Sun et al, 2019 (37)**	Single-center, China	2010-2015	To compare HRQoL after tailored RCT before surgery	5FU + RT/mFOLFOX6+ RT (nCRT group, n=132) vs. mFOLFOX6 alone (nCT group, n=88)	220	nCRT group: 88(66.7)<br>nCT group: 57(64.8)<br>N(%)	nCRT group :<br><5: 52(39.4)<br>5-10:73(55.3)<br>>10: 7(5.3)<br>nCT group:<br><5: 29(33)<br>5-10:55(62.5)<br>>10: 4(4.5)<br>N(%)	nCRT group:<br>56(27-77)<br>nCT group:<br>55(21-77)<br>Median(range)	EORTC-QLQ C30/CR29	40 mo (median fu after the end of the treatment)	Better global health status, role functioning, and social functioning, for the nCT group. Worse stool frequency, flatulence, fecal incontinence, sore skin and embarrassment for the RT group.
**Verweij et al, 2021 (38)**	Multicenter, Netherlands	N/A	To compare HRQoL after tailored RCT before surgery	Dose-escalated CRT (5x3 Gy boost + CRT) (Study group, n=51) vs. CRT alone (Control group, n=64)	115	Study group: 38 (74.5)<br>Control group: 47(73.4)<br>N(%)	Study group:<br>≤3 cm: 27(52.9)<br>3.1-5 cm: 8(15.7)<br>5.1-10: 16(31.4)<br>Control group:<br>≤3 cm: 36(57.1)<br>3.1-5 cm: 8(12.7)<br>5.1-10: 19(30.2)<br>N(%)	Study group:<br>64(26-75)<br>Control group:62(37-80)<br>Median (range)	EORTC-QLQ C30/CR29	3, 6, 12, 18, 24 mo after start treatment	No difference
**Erlandsson et al, 2021 (39)**	Multicenter, Sweden	1998-2013	To compare HRQoL after tailored RCT before surgery	5x5Gy RT plus surgery within 1 we (SRT, n=51) vs. 5x5Gy RT plus surgery after 4-8 we (SRT-delay, n=57) vs. 25x2Gy RT with surgery after 4-8 we (LRT-delay, n=61)	169	Three arm randomization<br>SRT group :33(64.7)<br>SRT-delay group :40(70.2)<br>LRT-delay group:38(62.3)<br>N(%)	N/A	Three arm randomization<br>SRT group:64(35-79)<br>SRT-delay group:63(41-83)<br>LRT-delay group:65(40-79)<br>Median(range)	EORTC QLC-C30	4-6y	No difference
**Kosmala et al, 2021 (40)**	Multicenter, Germany	2006-2010	To compare HRQoL after tailored RCT before surgery	Preoperative CRT followed by TME and postoperative CT (5FU/OX) (n=513) vs Preoperative CRT followed by TME and postoperative CT (5FU) (n=512)	1025	5FU/OX group: 356(69)<br>5FU group: 362(71)<br>N(%)	5FU/OX group:<br>0-5: 207(40)<br>>5-10:258(50)<br>>10:42(8)<br>No data: 6(1)<br>5FU group:<br>0-5: 173(34)<br>>5-10:278(54)<br>>10:57(11)<br>No data:4(1)<br>N(%)	5FU/OX group:<br>64(56-70)<br>5FU group:<br>64(56-70)<br>Median(IQR)	EORTC QLQ C30<br>EORTC QLQ CR38	After postoperative CT and during fu (1 and 3 y)	No difference

Table S2. Articles on pre- and/or post-CT and/or RT included in the review (continued)											
**Author, year (Ref.)**	**Study Design, Country**	**Duration of study**	**Population**	**Intervention**	**Number of Patients**	**Sex (M, %)**	**Distance from the av (cm)**	**Age (y)**	**Questionnaire(s)**	**Times of assessment**	**Key Results**
**Fokas et al, 2022 (41)**	Multicenter	3y	To compare HRQoL after tailored RCT before surgery	5FU, leucovorin and oxaliplatin before 5FU/OXA CRT (Group A, n=156) vs CRT before CT (Group B, n=150)	306	Group A: 106(68) Group B: 100(67) N (%)	Group A: 0-5:64(41) >5-10:67(43) >10: 15(10) Unknow:10(6) Group B: 0-5:62(41) >5-10:73(49) >10: 11(7) Unknow:4(3) N (%)	Group A: 60(11) Group B: 62(10) Mean(SD)	EORTC QLQ C30	1 and 3y	No difference
**Ganz et al, 2022 (42)**	Multicenter, USA	2004-2010	To compare HRQoL after after tailored RCT before surgery	5-FU + RT (n=277) vs. 5-FU + OXA + RT (n=266) vs. CAPE + RT (n=283) vs. CAPE + OXA + RT(286)	1112	Overall: 939(68.4) N(%)	N/A	Overall: 57.6(11.3) Mean(SD)	SF36, FACT-C	1, 5y after surgery	No difference
**Rouanet et al, 2022 (43)**	Multicenter, France	May 2011- Oct 2014	To compare HRQoL after after tailored RCT and induction high-dose chemotherapy.	Good responders after induction CT (n=30): surgery (Group A, n=11) or standard RCT plus surgery (Group B, n=19) vs. Poor responders after induction CT (n=103): Capecitabine (Group C, n=52) or standard RCT (Group D, n=51) before surgery	133	Group A:5(45.5) Group B:11(57.9) Group C:34(65.4) Group D:40(78.4) N(%)	N/A	Group A:66(44-78) Group B:63(39-75) Group C:61(22-82) Group D:62(22-80) Median(range)	EORTC QLQ-C30	1, 4, 8, 12, 24, 36, 48, and 60 mo	No difference
**Dijkstra et al, 2022 (44)**	Multicenter, Netherlands, Sweden, Spain, Slovenia, Denmark, Norway, and the USA.	2011-2026	To compare HRQoL after Short course RT, CT, TME vs CRT, TME and optional adjuvant CT in patients with LARC planned for surgery	Short course RT, CT, TME (Study group, n=243) vs CRT, TME and optional adjuvant CT (Experimental group, n=210)	453	Study group: 144(59.3) Control group: 99(40.7) N(%)	Study group : <5: 46(18.9) 5-10:102(42) >10: 79(32.5) Unknown: 16(6.6) Control group: <5: 50 (23.8) 5-10:73(34.8) >10: 71(33.8) Unknown: 16(7,6) N(%)	Study group: 63(55-68) Control group:62(54-69) Median (range)	EORTC QLQ C30 EORTC QLQ CR29	36 mo	No difference

Table S2. Articles on pre- and/or post-CT and/or RT included in the review (continued)											
**Author, year (Ref.)**	**Study Design, Country**	**Duration of study**	**Population**	**Intervention**	**Number of Patients**	**Sex (M, %)**	**Distance from the av (cm)**	**Age (y)**	**Questionnaire(s)**	**Times of assessment**	**Key Results**
**Dijkstra et al, 2022 (44)**	Multicenter, Netherlands, Sweden, Spain, Slovenia, Denmark, Norway, and the USA.	2011-2026	To compare HRQoL after Short course RT, CT, TME vs CRT, TME and optional adjuvant CT in patients with LARC planned for surgery	Short course RT, CT, TME (Study group, n=243) vs CRT, TME and optional adjuvant CT (Experimental group, n=210)	453	Study group: 144(59.3) Control group: 99(40.7) N(%)	Study group : <5: 46(18.9) 5-10:102(42) >10: 79(32.5) Unknown: 16(6.6) Control group: <5: 50 (23.8) 5-10:73(34.8) >10: 71(33.8) Unknown: 16(7,6) N(%)	Study group: 63(55-68) Control group:62(54-69) Median (range)	EORTC QLQ C30 EORTC QLQ CR29	36 mo	No difference
**Basch et al, 2023 (45)**	Multicenter, USA	June 2012- Dec 2018	To compare HRQoL after preoperative FOLFOX vs. preoperative 5FUCRT in patients with LARC planned for surgery	Preoperative FOLFOX (n=493) vs Preoperative 5FUCRT (n=447)	940	FOLFOX gorup:315(63.9) 5FU CRT group: 303(67.8) N(%)	N/A	FOLFOX group: 57(19-86) 5FU CRT group:58(25-84) Median (range)	EuroQoL EQ-5L	12 mo after surgery	Lower rates of fatigue and neuropathy and better sexual function in FOLFOX group
**Bascoul-Mollevi et al, 2023 (46)**	Multicenter, France	2012-2017	To compare HRQoL after nCT then CRT, TME and adjuvant CT vs. CRT, TME and adjuvant CT in patients with LARC planned for surgery	Neoadjuvant CT (mFOLFIRINOX) then CRT, TME and adjuvant CT (Study group, n=183) vs CRT, TME and adjuvant CT (Experimental group, n=187)	370	Study group: 118(64.5) Control group: 131 (70) N(%)	Study: ≤5 cm: 63(34.4) 6-10 cm: 92(50.3) >10: 28(15.3) Control: ≤5 cm: 73(39) 6-10 cm: 90(48.2) >10: 24(12.8) N(%)	Study group: 60(34-76) Control group:62(26-75) Median (range)	EORTC QLQ C30 EORTC QLQ CR29	1y, 2 y after treatment	No difference

LAR: Low Anterior Resection; HRQoL: Health Related Quality of Life; LARS: Low Anterior Resection Syndrome; CT: chemotherapy; RT: radiotherapy; RCT: radiochemotherapy; nCT: neoadjuvant CT; TME: Total Mesorectal Excision

Table S3. Articles on patient care strategies included in the review

**Table S3. Articles on patient care strategies included in the review (continued)**											
**Author, year (Ref.)**	**Study design, country study in**	**Duration of study**	**Population**	**Intervention**	**Number of Patients**	**Sex (M, %)**	**Distance from the av (cm)**	**Age (y)**	**Questionnaire(s)**	**Times of assessment**	**Results**
**Lee et al, 2013 (47)**	Sigle Centre, South Korea	Jul 2007- Sept 2011	To compare HRQoL after rehabilitation vs. conventional care in patients who had undergone laparoscopic LAR with defunctioning ileostomy	Rehabilitation (n=52) vs. Conventional care (n=46)	98	Rehabilitation group: 36(70.0) Conventional care group: 28(62.8) N(%)	Rehabilitation group: 4(1.5) Conventional care group: 4.7(2) Mean (SD)	Rehabilitation group: 61.2(10.8) Conventional care group: 61.7(10.8) Mean(SD)	SF 36	30-day after the operation	No difference
**Moug et al, 2019 (48)**	Single Centre, UK	Aug 2014- Mar 2016	To compare HRQoL after prehabilitation vs. conventional care in patients planned for nCRT followed by potentially curative surgery	Prehabilitation (n=24) vs. Conventional care (n=24)	48	Prehabilitation group: 18(75) Conventional care group: 13(54.1) N(%)	N/A	Prehabilitation group: 65.2(11.4) Conventional care group: 66.5(9.6) Mean(SD)	EORTC-QLQ CR29	1-2 w before the operation	No difference
**Cuicchi et al, 2020 (49)**	Sigle Centre, Italy	Jan 2015-Oct 2015	To compare HRQoL after Percutaneous Posterior Tibial Nerve Stimulation vs. Medical Therapy alone in patients who underwent nCRT and LAR for cancer with LARS score ≥ 21 and ileostomy closed at least 18 mo earlier	Percutaneous Posterior Tibial Nerve Stimulation (Study group, n=6) vs. Medical Therapy alone (Control group, n=6)	12	Study group: 3(50) Control group: 4(66.7) N(%)	Study group: 4.2(1.5) Control group: 3.2(1) Mean (SD)	Study group: 62.5(50-75) Control group: 71.5(56-79) Median (Range)	EORTC-QLQ-C30	1 yr	No difference

Table S3. Articles on patient care strategies included in the review (continued)											
**Author, year (Ref.)**	**Study design, country study in**	**Duration of study**	**Population**	**Intervention**	**Number of Patients**	**Sex (M, %)**	**Distance from the av (cm)**	**Age (y)**	**Questionnaire(s)**	**Times of assessment**	**Results**
**Yoon et al, 2020 (50)**	Single Centre, South Korea	Jun 2016-Mar 2018	To compare HRQoL after probiotic vs. conventional care in patients undergoing ileostomy closure after LAR for RC	Probiotic (*Lactobacillus plantarum*) (Study group, n=17) vs. conventional care (Control group, n=19)	36	Study group: 11(70) Control group: 12(57) N(%)	Study group: <5 cm: 0(0.0) 5 cm ≤ tumor < 10 cm: 15(78.9) 10 cm ≤ tumor < 20 cm: 4(21.1) Control group: <5 cm: 6(35.3) 5 cm ≤ tumor < 10 cm: 9(52.9) 10 cm ≤ tumor < 20 cm: 2(11.8) N(%)	Study group: 62.1(9.4) Control group: 57.2(12.2) Median (IQR)	EORTC-QLQ CR29 EORTC-QLQ-C30	1 w and 3 w following ileostomy reversal,	No difference
**Morielli et al, 2021 (51)**	Single Centre, USA	N/A	To compare HRQoL after exercise vs conventional care in patients undergoing nCRT	Exercise (Study group, n=16) vs. Conventional care (Control group, n=16)	32	Overall: 67 %	N/A	Overall: 57(12) Mean (SD)	EORTC-QLQ CR29 EORTC-QLQ-C30	Post nCRT, Pre-surgery	Worst QoL in the study group (diarrhea p=0.03 and social embarrassment p=0.003)
**Su et al, 2021 (52)**	Multicenter, China	Jan 2015-Dec 2015	To compare HRQoL after Continuing Care Bundle vs conventional care in patients with temporary ileostomy after LAR	Continuing Care Bundle (Study group, n=50) vs. conventional care (Control group, n=57)	107	Study group:35(70) Control group:32(56.1) N(%)	Study group: ≤5 cm: 4(8.0) 6-10 cm: 24(48) >10: 22(44) Control group: ≤5 cm: 7(12.0) 6-10 cm: 27(47) >10: 23(40) N(%)	N/A	Stoma-QOL	4 and 12 weeks after surgery	Better QoL in the study group (p<0.001)
**Asnong et al, 2022 (53)**	Multicenter, Belgium	N/A	To compare HRQoL after PFMT vs. conventional care in patients after	PFMT(Study group, n=50) vs Conventional Care (Control group, n=54)	104	Study group: 36 (72) Control group: 35(64.8)	Study group: ≤5 cm: 29(58.0) 6-10 cm: 14(28) >10: 7(14)	Study group: 58.8(12.7) Control group: 57.1(10.9)	SF-12	1, 4, 6 and 12 mo after the operation	No difference

			LAR and a minimal LARS score of 21/42 at 1 mo after surgery	*PFMT: Pelvic Floor Muscle Training*		N(%)	Control group:<br>≤5 cm: 31(59.3)<br>6-10 cm: 16(29.6)<br>>10: 7(11.1)<br><br>N(%)	Mean (SD)			

Table S3. Articles on patient care strategies included in the review (continued)

**Author, year (Ref.)**	**Study design, country study in**	**Duration of study**	**Population**	**Intervention**	**Number of Patients**	**Sex (M, %)**	**Distance from the av (cm)**	**Age (y)**	**Questionnaire(s)**	**Times of assessment**	**Results**
**Van der Heijden et al, 2022 (54)**	Multicenter, Germany	Oct 2017- Mar 2020	To compare HRQoL after Pelvic Floor Rehabilitation vs conventional care in patients after LAR	Pelvic Floor Rehabilitation (Study group, n=44) vs. Conventional Care (Control group, n=51)	95	Study group: 26(59.1)<br>Control group: 33 (64.7)<br><br>N(%)	Study group: 5.7(2.5<br>Control group: 5.4(2.3)<br><br>Mean (SD)	Study group: 63(12)<br>Control group: 63(17)<br><br>Median (IQR)	EORTC-QLQ-CR29	3 mo after surgery without temporary stoma or 6 we after stoma closure	No difference (* only better HRQoL in patients suffering from fecal incontinence)
**Pieniowskui et al, 2023 (55)**	Multicenter, Sweden	May 2016- Nov 2019	To compare HRQoL after TAI vs conventional care in patients after LAR and a LARS score of 21/42 at 6 mo after surgery	TAI (Study group, n=22) vs Conservative Treatment (Control group, n=23)<br><br>*TAI: Transanal Irrigation*	45	Study: 11(50)<br>Control: 14(60.8)<br><br>N(%)	Study: 10(2)<br>Control: 10(3)<br><br>Mean (SD)	Study: 65(10)<br>Control: 64(13)<br><br>Mean(SD)	EORTC-QLQ-C30	12 mo	Better QoL in the intervention group
**Kim et al, 2023 (56)**	Single Centre, Korea	N/A	To compare HRQoL after bowel function improvement program vs. conventional care	Bowel Function Improvement Program (Study group, n=21) vs. Conventional Care (Control group, n=21)	42	N/A	Study group:<br>≤5 cm: 7(33.3)<br>6-10 cm: 9(42.9)<br>>10: 5(23.8)<br>Control group:<br>≤5 cm: 4(19.1)<br>6-10 cm: 15(71.4)<br>>10: 2(9.5)<br><br>N(%)	Study group: 58.10 (7.76)<br>Control group: 57.05 (8.9)<br><br>Mean(SD)	EORTC-QLQ-CR29	3mo after surgery	No difference

LAR: Low Anterior Resection; HRQoL: Health Related Quality of Life; LARS: Low Anterior Resection Syndrome; TME: Total Mesorectal Excision

Figure S1. Risk of Bias Assessment (RoB 2) for Surgical Intervention Studies.

Study ID	D1	D2	D3	D4	D5	Overall
Andersson et al.	-	+	+	!	+	!
Russell et al.	-	!	!	!	+	-
Okkabaz et al.	+	!	!	+	+	-
Musters et al	+	+	+	+	+	!
Gadan et al.	+	!	!	+	+	!
Jayne et al.	+	!	!	+	+	!
Kim et al.	+	!	!	+	+	!
Park et al.	+	!	!	+	+	!
Parc et at.	+	!	!	+	+	!
Ribi et al.	+	!	!	+	+	!
Gavaruzzi et al.	+	!	!	+	+	!
Bach et al	+	!	!	+	+	!
Elsner et al.	+	!	!	+	+	!
Dulskas et al.	+	!	!	+	+	-
Ellebaek et al	+	!	!	+	+	!
Ahmadi-Amoli et al.	+	!	!	+	+	!

\+ Low risk
! Some concerns
\- High risk

D1 Randomisation process
D2 Deviations from the intended interventions
D3 Missing outcome data
D4 Measurement of the outcome
D5 Selection of the reported result

Figure S2. Risk of Bias Assessment (RoB 2) for Pre- and/or Post-Chemotherapy and/or Radiotherapy Studies.

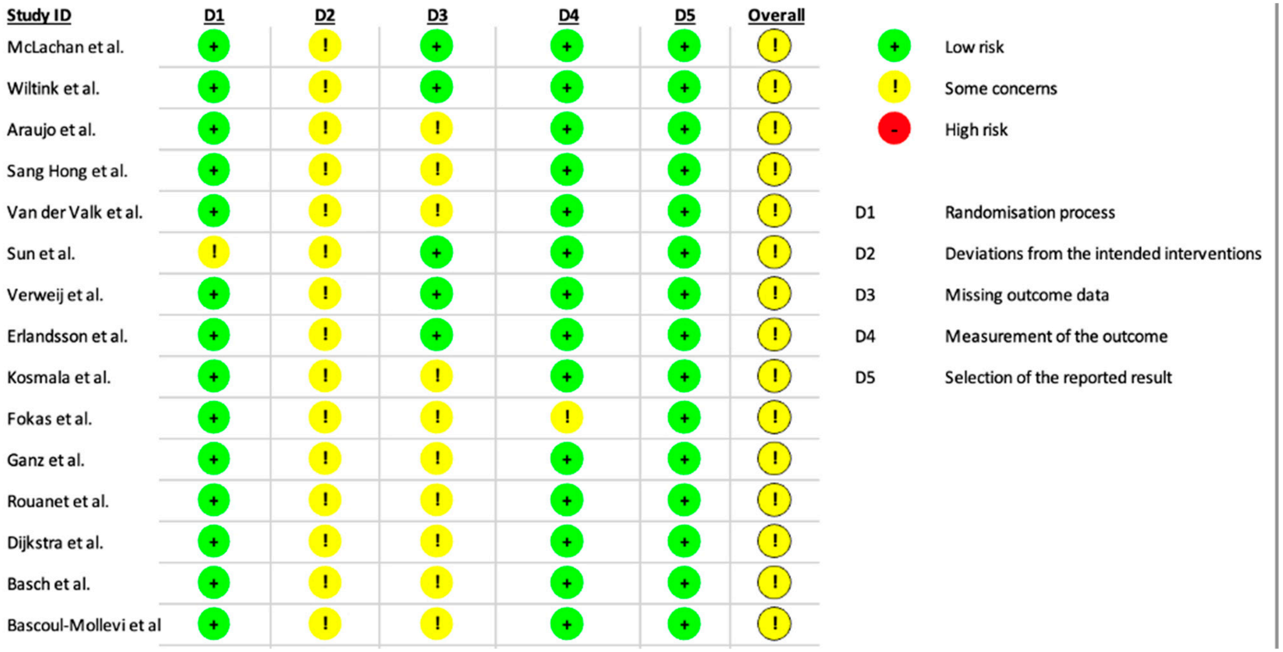

Figure S3. Risk of Bias Assessment (RoB 2) for Patient Care Strategy Studies.

Study ID	D1	D2	D3	D4	D5	Overall
Lee at al.	+	!	!	!	+	!
Moug et al.	+	!	!	+	+	!
Cuicchi et al.	!	!	!	+	+	!
Yoon et al.	+	+	+	+	+	+
Morielli et al.	+	!	!	+	+	!
Su et al.	+	!	!	+	+	!
Asnong et al.	+	!	+	+	+	!
Van der Heijden et al	+	!	!	+	+	!
Pieniowskui et al.	+	!	!	+	+	!
Kim et al.	+	!	+	+	+	!

+ Low risk
! Some concerns
- High risk

D1 Randomisation process
D2 Deviations from the intended interventions
D3 Missing outcome data
D4 Measurement of the outcome
D5 Selection of the reported result
